# The Development of a Bi-Lingual Assessment Instrument to Measure Agentic and Communal Consumer Motives in English and French

**DOI:** 10.3389/fpsyg.2016.01198

**Published:** 2016-08-11

**Authors:** Mike Friedman, Anne-Laure Bartier, Josh Lown, Christopher J. Hopwood

**Affiliations:** ^1^Department of Marketing, Louvain School of Management, Catholic University of LouvainLouvain-la-Neuve, Belgium; ^2^Department of Marketing, EPHEC Business SchoolLouvain-la-Neuve, Belgium; ^3^Department of Psychology, Michigan State UniversityEast Lansing, MI, USA

**Keywords:** agency, communion, motivations, individual differences, bi-lingual scale development, consumer psychology

## Abstract

Consumer behavior is driven, in part, by the degree to which goods and services appeal to underlying motives for agency and communion. The purpose of this research was to develop a brief individual differences measure of these motivations for use in behavioral research and theoretical and applied consumer psychology and marketing studies. We employed a bi-lingual scale development procedure to create the 10-item Agentic and Communal Consumer Motivation Inventory (ACCMI) in English and French. Two studies show that the ACCMI is language invariant, demonstrates convergent and discriminant validity with consumer, motivational, and interpersonal constructs, and predicts evaluations of products described in agentic and communal terms, respectively, in both languages. The general conclusion of this research is that agency and communion provide a useful framework for understanding and studying consumer buying motivations. Discussion focuses on the relevance of motivational factors for studying human behavior and the applied utility of the ACCMI.

## Introduction

Many factors affect why and how consumers choose products, brands, and services. A major focus of previous research has been on consumer information processing and judgment making (Bettman, 1979; Wyer, 2008). This work has provided a great deal of knowledge about how consumers perceive and choose products, brands, and services. Consumer motivations represent an important but often-neglected lens through which to understand and to study consumer psychology (Pham, 2013). Indeed, motivational factors such as the feelings associated with products or the meanings that are attributed to them are likely to exert a powerful influence on certain aspects of consumer behavior (Dichter, 1964). Understanding individual differences in these motivations, in addition to general cognitive processes associated with consumption and the perceived value of a product's attributes, would provide a fuller picture of why certain consumers prefer certain types of products and brands. Such an understanding could contribute, in turn, to assessment tools that could facilitate research on consumer psychology and be of use to companies who wish to more effectively align marketing practices and communications with their customers' motivations to acquire goods and services.

### Agency and communion

Our approach to understanding consumer motivations was guided by research showing that *agency* and *communion* structure the broadest level of human values and motives across cultures (Locke, 2000; Trapnell and Paulhus, 2012), implying that these dimensions provide a viable framework for studying consumer motivations, as well. Agentic values involve the desire to be a differentiated individual, separate and autonomous from others, and to assert and expand the self (Wiggins, 1991; Helgeson, 1994; Abele and Wojciszke, 2007; Abele et al., 2008). Communion involves the desire to be part of a larger social entity or community and to establish close relationships or social connections with others (Wiggins, 1991; Helgeson, 1994; Abele and Wojciszke, 2007; Abele et al., 2008). Notably, this framework has proven useful for conceptualizing many social and psychological constructs in addition to values, such as traits (Markey and Markey, 2009), problems (Alden et al., 1990), strengths (Hatcher and Rogers, 2009), and sensitivities (Hopwood et al., 2011), and has served as the basis for the development of a number of assessment tools (Markey and Markey, 2009; Hopwood et al., 2011; Trapnell and Paulhus, 2012).

These themes would seem readily applicable to consumer motives. For instance, people could be influenced to buy products based in part on the degree to which the product would satisfy agentic (e.g., feeling autonomous and free) or communal (e.g., feeling close and connected to others) motives. It appears that companies and applied marketers share this intuition, given that agentic and communal motives are used to advertise or describe the benefits of many different products and brands. For example, both Mountain Dew and Coca-Cola are similarly high-sugar, carbonated, caffeinated, beverages. Despite the similarities of the actual products, these brands' marketing actions differentiate their offers through motivational appeals, with agency figuring prominently in Mountain Dew's campaign prompting consumers to “do the Dew” (by achieving, excelling and triumphing over nature in extreme sports), and communion described as a primary benefit in Coca-Cola's “share a Coke” advertisements.

Previous research provides strong hints about the importance of agency and communion in motivating consumer preferences and behaviors. For example, agency is clearly reflected in studies of consumer uniqueness (e.g., Tian et al., 2001), which aim to understand the ways in which consumers pursue differentiation from others through “the acquisition, utilization, and disposition of consumer goods for the purpose of developing and enhancing one's self-image and social image” (p. 52). Previous research has shown that, under certain circumstances, consumers can use luxury brands in an agentic fashion to distinguish themselves from others (Han et al., 2010), and studies of youngsters have shown that social motivations such as distinction from or connection to others can be important drivers of consumption behavior (Baker and Gentry, 1996; Chaplin and John, 2005). Furthermore, adult consumers with unconventional professions (e.g., tattoo/body-piercing artists) or hobbies (e.g., owning low-rider cars), for example, are higher in consumer need for uniqueness than members of the general population (Tian et al., 2001).

Another strand of relevant research focuses on consumer conformity (Bearden and Rose, 1990; Ruvio, 2008), defined as the tendency to conform to others' preferences for the products and brands one prefers, uses or buys (Bearden and Rose, 1990). Consumers who score high on measures of conformity are more likely to yield to interpersonal influence in their consumption behavior, preferring or acquiring products that are endorsed or used by others in their social environment (Bearden and Rose, 1990; Mandrik et al., 2005).

Connections between agency with uniqueness and communion with conformity seem intuitive, but these connections have not been established empirically. Conceptualizing consumer motivations using agency and communion as a broad framework would promote the simultaneous assessment of a range of potentially applicable motives and more generally connect research on motivated buying behavior to broader themes in the social science literature.

In the current research we sought to test whether agency and communion represent motives underlying consumer behavior. A second goal was to create a measure to assess both agentic and communal consumer motives, which could be used for research in consumer psychology, applied marketing practice, and basic research on motivations in the buying domain.

### Cross-cultural scale development

Given our desire for a measure that is as broadly applicable as possible, we created and validated the instrument in both English and French simultaneously. This procedure is different than most scale development processes in which instruments are developed in a single language (an imposed etic approach, Berry, 1989; Craig and Douglas, 2005; De Jong et al., 2009) and then extended to others via back-translation (Brislin, 1970). This typical scale development procedure results in scales that are optimized for use within a particular language, but whose functioning in another language is not guaranteed. Indeed, multi-group confirmatory factor analytic methods which are used assess structural invariance across scale language versions (Vandenberg and Lance, 2000; Nye et al., 2008) commonly reveal poor fit when assuming identical structure for the scale in its original language and translated versions (Nye et al., 2008; Church et al., 2011).

Therefore, in this study we developed the instrument in French and English simultaneously. This approach was supported by research showing that agency and communion provide a common structure for values and traits across different cultures and languages (Wiggins, 1991; Abele et al., 2008; Trapnell and Paulhus, 2012). This allowed us to consider structural (McCrae et al., 1996; Nye et al., 2008) and predictive (Oishi and Roth, 2009) equivalence in item selection. The hope was to create a consumer motivation scale that is structurally and functionally equivalent across two languages, and thus likely to tap basic underlying dimensions of human (or, at least Western) buying behavior rather than culturally specific patterns.

In summary, our goals were to test whether agency and communion represent motives underlying consumer behavior and to develop an instrument to measure those motives among English and French speakers. In Study 1, we describe the development and refinement of the *Agentic and Communal Consumer Motivation Inventory* (ACCMI) scale items using data from two large English and French-speaking samples. We use factor analytic techniques to refine the measure and test structural invariance across language versions, in addition to examining temporal stability and convergent and discriminant validity. In Study 2, we confirm the structural invariance of the ACCMI across language versions and study the ability of the ACCMI to predict consumer preferences in both languages.

## Study 1

The goal of Study 1 was to develop and refine a set of items to measure agentic and communal consumer motivations in both English and French. In addition to item creation and refinement, we sought to assess convergent and discriminant relationships of our scale with other theoretically relevant measures, and to assess the temporal stability of the ACCMI. Due to practical considerations, convergent and discriminant validity were tested in the English language sample and temporal stability was tested in the French language sample.

We hypothesized that agentic consumer motivations would evidence moderate positive relationships with other agentically-related measures. Specifically, we expected moderate positive relationships between our measure of agentic consumer motivations and (1) existing measures of agentic values and traits and (2) an existing measure of consumer motivations: the consumer need for uniqueness. We also hypothesized that communal consumer motivations would evidence moderate positive relationships with other communally-related measures. Specifically, we expected moderate positive relationships between our measure of communal consumer motivations and (1) existing measures of communal values and traits and (2) existing measures of consumer and social motivations: conformity motivations and the need to belong.

### Method

#### Item development

Given the importance of agency and communion for structuring a range of social behavior, values, and motivations, and the fact that existing research on consumer psychology has studied constructs which seem to be subsumable within these two meta-concepts, we started the item development process with a review of two primary bodies of literature. The bodies of literature were chosen based on their theoretical relevance to the research questions and to their importance and grounding within the research domain (psychology, marketing, and consumer behavior). The first body of literature focused on agency and communion as discussed theoretically (e.g., Wiggins, 1991; Abele and Wojciszke, 2007) and as employed empirically (e.g., Abele et al., 2008; Trapnell and Paulhus, 2012) to develop measurement instruments in other domains (e.g., traits, values, etc.). The second body of literature focused on motivational and consumer psychology constructs which evidence parallels with agency and communion. Constructs which parallel agency include uniqueness theory (e.g., Snyder and Fromkin, 1977), independent self-construal (e.g., Markus and Kitayama, 1991; Singelis, 1994), desire for consumer uniqueness and differentiation from others (e.g., Tian et al., 2001; Lynn and Snyder, 2002). Constructs which parallel communion include the need to belong (e.g., Baumeister and Leary, 1995), interdependent self-construal (e.g., Markus and Kitayama, 1991; Singelis, 1994) and the desire for consumer conformity (e.g., Bearden and Rose, 1990). In this first step, we sought to extract key themes from the existing literature on these topics, in order to write items that comprehensively sample the kinds of agentic and communal motives thought to be relevant to consumer behavior.

Following this literature review, first versions of the items were developed by two researchers. A native English speaker developed items in English, while a native French speaker developed items in French. The literature review was jointly conducted by these two researchers, with theory and previous research being thoroughly discussed prior to and throughout the item development process. However, the process of item development for each language was carried out independently by each researcher.

This process resulted in 132 items. A first culling was done by the item developers to determine redundant and overlapping items within the item sets for each language. All items were then back-translated to compile a master list, which included all items in both their English and French versions. Two separate groups of researchers evaluated this initial item pool for content, readability, and fit with the theoretical constructs. A group of native English speakers evaluated the English versions of the items, while a group of native French speakers evaluated the French versions of the items. Items that were judged as not fitting the content area or as being linguistically problematic were deleted. After item culling, 90 items were retained for first testing, with an approximately equal number originating in French (*n* = 40) and English (*n* = 50) and an approximately equal number of agency (*n* = 48) and communion (*n* = 42) items.

#### Participants

Participants were undergraduate students in an Anglophone and a Francophone university. All participants received partial course credit for their participation. Anglophone participants were 987 students at a large Midwestern university in the United States (319 males, 656 females, 12 did not report gender), with a mean age of 19.65 years (*SD* = 1.81). Francophone participants were 905 individuals (379 male, 496 female, 30 did not report gender), with a mean age of 20.43 years (*SD* = 2.29), at a large public French-speaking university in the Walloon region of Belgium.

Participants in the US completed all of the questionnaires described below. Participants in Belgium completed only the first version of the ACCMI. A subset of the Belgian sample (N = 123), completed the full 90 item scale at two different time points, separated by 3 weeks.

##### Measures

**First version of the ACCMI**. Participants indicated the extent to which it is important to them that the products they buy, in general, help them to attain agentic (e.g., to be unlike others) or communal (e.g., to seek connection with others) motives. Participants evaluated each of the 90 preliminary items on a 6-point Likert scale from 1 (“not important to me”) to 6 (“extremely important to me”).

*Consumer measure related to agency*. **Consumer Uniqueness Scale (CUS; Tian et al., 2001)**. The CUS consists of 36 items that measure the extent to which consumers seek differentiation from others through the acquisition and use of consumer goods. Responses are given on a 5-point Likert-type scale, from 1 (“strongly disagree”) to 5 (“strongly agree”). An example item is “I collect unusual products as a way of telling people I'm different.” Internal consistency was 0.95.

*Consumer measures related to communion*. **Attention to Social Comparison Information (ATSCI; Lennox and Wolfe, 1984)**. The ATSCI consists of 13 items which measure the extent to which individuals are aware of the reactions of others to their behavior and the extent to which they are concerned about or sensitive to the nature of those reactions. The ATSCI has been used in previous research to index consumer conformity motivations (Bearden and Rose, 1990; Mandrik et al., 2005; Ruvio, 2008). An example item is “I usually keep up with clothing style changes by watching what others wear.” Responses are given on a 7-point Likert scale from 1 (“always false”) to 7 (“always true”). Internal consistency was 0.82.

**Need to Belong (NTB; Leary et al., 2013)**. The NTB scale measures the extent to which individuals desire to form and maintain enduring interpersonal attachments. Responses are given on a 5-point Likert scale, from 1 (“strongly disagree”) to 5 (“strongly agree”). An example item is “I try hard not to do things that will make other people avoid or reject me.” Internal consistency was 0.80.

*Measures of agentic and communal values and traits*. **Agentic and Communal Values Scale (ACV; Trapnell and Paulhus, 2012)**. The ACV measures overall agentic and communal motivations. Participants evaluate 24 different values on a 9-point Likert scale from 1 (“not important to me”) to 9 (“highly important to me”) on the extent to which each value serves as a guiding principle in their lives. Twelve items assess agentic motivations [e.g., autonomy (independent, free of others' control)] and 12 items assess communal motivations [e.g., compassion (caring for others, displaying kindness)]. Internal consistencies for the agentic and communal scales were 0.83 and 0.88, respectively.

**International Personality Item Pool-Interpersonal Circumplex (IPIP-IPC; Markey and Markey, 2009)**. This scale consists of a series of personality items which can be used to measure agentic and communal traits. Specifically, participants rate themselves on a series of 32 traits (e.g., “am interested in people”) using a 5-point Likert scale from 1 (“very inaccurate”) to 5 (“very accurate”). Omnibus scores for agency (termed “dominance” in the IPIP-IC framework) and communion (termed “warmth” in the IPIP-IC framework) were calculated for each participant using the scoring algorithm provided by Markey and Markey (2009). Internal consistencies were calculated using an equation to compute reliabilities of weighted sums (Nunnally and Bernstein, 1994, Equations 7–17), and were 0.86 and 0.85, respectively, for the agentic and communal scales.

*Ethics statement and data availability*. The research study was approved in the US by the Institutional Review Board at Michigan State University, No. 12-715e, 2012. In Belgium there is no legal requirement to obtain approval from an institutional review board (IRB) for non-clinical research studies. Two authors of the current paper conducted this research while employed at a Belgian university (Catholic University of Louvain) in which no IRB existed at the time when the data were collected. Belgian participant data were anonymized prior to author access and analysis and no identifying, personal, or health related information was collected. Written informed consent was obtained in the US, and verbal informed consent was obtained in Belgium.

The data from this study are available freely and without restriction at: osf.io/yd3uk.

## Results

### Principal components analysis

A series of Principal Components Analyses (PCA) were conducted in order to (1) determine whether agency and communion dimensions could be identified in the ACCMI items and (2) identify candidate items that could be culled in the process of developing an efficient measurement tool. First, the 90 items of the ACCMI were subjected to PCA separately in each language. As would be expected in a PCA with 90 item-level variables, there were a number of components with eigenvalues above 1 in both languages. We focused on those components that explained a sizeable percentage of the variance and which had a large number of specific indicators (Floyd and Widaman, 1995).

The first two components explained 38 and 39% of the item variance in the English and French language versions, respectively. These two components were rotated to be orthogonal via the Varimax procedure, whereupon pattern coefficients clearly suggested agentic (e.g., “be original”) and communal (e.g., “seek connections with others”) themes. There were at least 24 items with strong and specific pattern coefficients for each component in both languages, suggesting that agentic and communal dimensions were strongly represented in our original item pools. Furthermore, Pearson congruence coefficients across languages were 0.92 for the agency dimension and 0.85 for the communion dimension, suggesting that agency and communion were characterized similarly across the English and French language versions.

The third component had eigenvalues of 4.83 and 4.04 in the English and French language versions, respectively. Factors beyond the third had comparably low eigenvalues, had dissimilar content across language versions, and were poorly represented in the item pool and thus were not considered further. We extracted three components and rotated them using Varimax to explore the third dimension further. Examination of pattern coefficients suggested a dimension involving motives toward conformity and fitting in (e.g., “act like others,” “be similar to others”). Unlike the agency and communion dimensions, only 5 items had strong and specific pattern coefficients for this component. Agency and communion dimensions from this analysis again exhibited strong congruence coefficients across languages (0.91 and 0.79, respectively), whereas the congruence for this third “conformity” component was somewhat lower (0.70).

We carefully considered retaining this third dimension for further analysis. However, several factors led us to focus on the first two dimensions. First, we anticipated identifying agency and communion dimensions given that (1) these two factors offer a viable structure for human motives and social behaviors more generally and (2) consumer psychology focuses on constructs that are closely related to these themes (e.g., need for uniqueness). By focusing on these two primary dimensions we retain a tight linkage between our results and the theory that frames our research questions. Second, one of our main goals was to develop an instrument that could be used to identify consumer motives in both English and French. Given that we were only able to identify five candidate items for this third dimension in our item pool, and that the dimension was less similar across English and French solutions than agency and communion, we were concerned about our ability to construct a reliable and robust bi-lingual “conformity” scale from these items. Third, a regression-based component score was unable to provide incremental information over the first two (agency and communion) factors when regressed upon criterion variables in the English-speaking sample (see results related to the consumer agency and communion scales below). In summary, while we regard conformity as a potentially useful dimension of consumer behavior worthy of further research, as discussed below, we focused upon the agency and communion dimensions in the research presented subsequently.

We next returned to the 2-factor PCA with Varimax rotation to identify the most robust indicators of the agency and communion dimensions. We retained items for further analysis if they were among the top 30 highest loading items in at least one language, had a relatively large (>|0.30|) loading on the same component in both languages, and had a relatively small (<|0.20|) cross-loading on the other component in both languages. This process led us to retain 24 agency and 32 communion items for the next step.

We next conducted a PCA using these items, in order to further refine the scale and determine the items that best represented the agentic and communal dimensions. The components with the highest eigenvalues in each language again clearly represented agentic and communal themes and explained 45% of the variance in both the English and French language versions. Components were again rotated to be orthogonal. Items were retained for the next step if they had a strong loading (>|0.45|) on the same factor in both languages and no cross-loadings on the other factor >|0.20|in either language. Based on this examination, 17 agency and 18 communion items were retained.

### Confirmatory factor analysis

Multi-group confirmatory factor analysis (MGCFA) was used to examine structural equivalence of the retained items across language versions. A first test of configural equivalence was conducted using the 35 items selected via the PCAs. This analysis was used to examine similarities for item loadings, intercepts, and variances across languages.

The item refinement at this stage was an iterative process, and proceeded using the following two techniques. First, the modification indices were inspected; we found that items with similar content (e.g., “belong” and “be accepted by others” to measure communion motivations) evidenced high covariance with one another. Instead of incorporating the co-variances explicitly in the model, we chose one representative item for each set of co-varying items and eliminated the other(s) from the scale.

Second, we used the model of configural equivalence to determine the extent to which each of the 35 items evidenced between-language discrepancies in the model estimates of their standardized loadings, intercepts, and variances. For each item, we created an aggregate score of between-language model fit based on the correspondence between the estimates for the loadings, intercepts, and variances for each item.

Based on these two measures, we selected a final set of 10 items for which inter-item co-variation was minimized and the correspondence between model estimates of loadings, intercepts and variances were maximized across languages. The final scale contained 5 items measuring agentic consumer motivations, and 5 items measuring communal consumer motivations. We then proceeded to a two-step MGCFA to determine structural invariance of the 10-item scale across language versions (Stark et al., 2006; Nye et al., 2008). All items for both scales were evaluated in the same model. In the first step, we tested for configural invariance, constraining the factor structure to be equal across language groups. In the second step, we tested metric (constraining the factor loadings to be equivalent across language versions) and scalar invariance (constraining intercepts to be equivalent across language versions) simultaneously.

**Figure 1:**
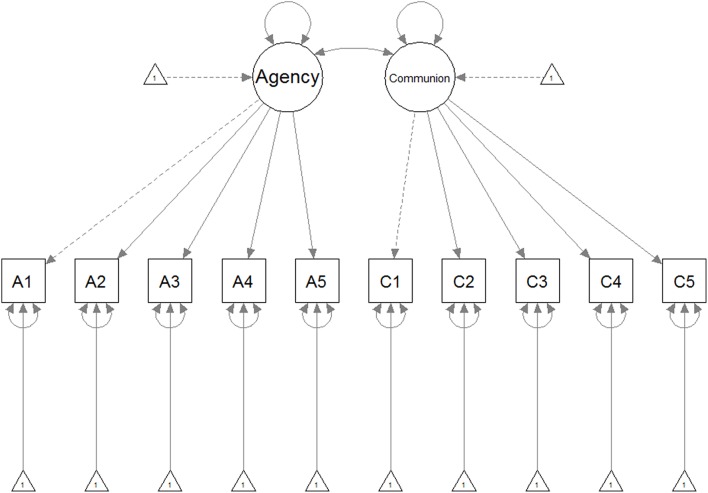
**Schematic overview of the structural model fit in Studies 1 and 2**.

For the analysis presented below, we used the Lavaan R package (Rosseel, 2012). Robust MLM estimation was used to evaluate the models. The fit indices of these models are displayed in Table 1. The factor loadings from the metric/scalar invariance model are displayed in Table 2. A schematic overview of the structural model is depicted in Figure 1. The covariance between the latent agentic and communal factors was 0.23 in the English and 0.22 in the French version of the ACCMI. A test for discriminant validity showed that the average variance extracted (AVE) values for the Agency dimension were 0.51 and 0.54 in the English and French language versions, while the AVE values for the Communion dimension were 0.52 and 0.53 in the English and French language versions. These values are in all cases larger than the squared correlation between the latent factors of these dimensions (0.11 and 0.09 for the English and French language versions, respectively).

**Table 1:** **Model fit for MGCFA analyses**.

**Model**	**χ^2^**	**df**	**χ^2^/df**	**CFI**	**TLI**	**RMSEA**
**STUDY 1**						
Configural invariance	195.53	68	2.88	0.98	0.97	0.05
Metric/Scalar invariance (equal loadings + intercepts)	374.53	84	4.46	0.95	0.95	0.06
**STUDY 2**						
Configural invariance	170.7	68	2.51	0.97	0.96	0.067
Metric/Scalar invariance (equal loadings + intercepts)	259.91	84	3.09	0.94	0.94	0.079

**Table 2:** **ACCMI items and factor loadings for metric/scalar invariance (Model 2)**.

**English version**	**French version**	**Factor loading Study 1**	**Factor loading Study 2**
**AGENTIC CONSUMER MOTIVATIONS**				
Be unlike others	ne pas ressembler aux autres	1.00	1.00
Stand out from others	me démarquer des autres	1.02	1.15
Be original	être original(e)	0.99	0.90
Be different	être différent(e) des autres	1.10	1.10
Be uncommon	être hors du commun	1.02	0.95
**COMMUNAL CONSUMER MOTIVATIONS**				
Belong	m'intégrer	1.00	1.00
Seek unity with others	rechercher l'union avec les autres	1.33	1.20
Seek connection with others	rechercher une connexion avec les autres	1.40	1.16
Seek harmony with others	rechercher l'harmonie avec les autres	1.35	0.94
Pay attention to others	être attentif(ve) aux autres	1.29	0.95

On the whole, these analyses suggest that the ACCMI is structurally invariant across language versions. Internal consistencies for the Agentic Consumer Motivations scale in the English and French versions were 0.84 and 0.86, respectively. Internal consistencies for the Communal Consumer Motivations scale for the English and French versions were 0.84 and 0.85, respectively. The final scale items in both English and French are presented in Table 2, and the full ACCMI questionnaires are presented in Appendices A, B.

### Temporal stability

A subset of the Francophone sample (*N* = 123, 48 male, 75 female, mean age = 20.35, *SD* = 2.08), completed the full 90 item scale at two different time points, separated by 3 weeks. Internal consistencies for the 5-item Agentic and 5-item Communal Consumer Motivation scales were 0.86 and 0.85, respectively, at Time 1 and 0.91 and 0.91, respectively, at Time 2. The test-retest correlations for the 5-item Agentic and 5-item Communal Consumer Motivation scales were 0.77 and 0.79, respectively.

### Convergent and discriminant validity

We conducted two sets of analyses to investigate the convergent and discriminant validity of the ACCMI. We first examined bivariate associations between the Agentic and Communal Consumer Motivation scales (calculated by summing all 5 items for each dimension) with the validation measures of agentic and communal constructs (e.g., CUS, ACV, etc.). Because we observed a correlation between the Agentic and Communal Consumer Motivation scales (*r* = 0.28, *p* < 0.001), we also calculated the partial correlations of the ACCMI scales by regressing each validation measure on the two ACCMI scales simultaneously. The partial correlations describe the relationship between each ACCMI scale (e.g., Agentic Consumer Motivations) and the validation measures, controlling for the other ACCMI scale (e.g., Communal Consumer Motivations). The results of these analyses are summarized in Table 3. Overall, both the Agentic and Communal Consumer Motivations scales converged well with measures of their respective domains, and were also reasonably discriminating, particularly in terms of partial correlations. Specifically, Agentic Consumer Motivations was related to consumer uniqueness and agentic values and traits whereas Communal Consumer Motivations was related to attention to social comparison information, need to belong, and communal values and traits.

**Table 3:** **Convergent and discriminant validity of the ACCMI scales, Study 1**.

**Consumer motivations**	**Correlations**		**Partial correlations**	
	**Agentic**	**Communal**	**Agentic**	**Communal**
**AGENTIC MEASURES**				
Consumer uniqueness	0.48^**^	−0.07^*^	0.53^***^	−0.22^***^
Agentic values	0.30^**^	0.19^**^	0.27^***^	0.11^**^
Agentic traits	0.22^**^	0.02	0.23^***^	−0.04
**COMMUNAL MEASURES**				
Attention to social comparison information	−0.09^**^	0.31^**^	−0.18^***^	0.36^***^
Need to belong	−0.11^**^	0.38^**^	−0.23^***^	0.44^***^
Communal values	0.16^**^	0.33^**^	0.08^*^	0.30^***^
Communal traits	0.08^*^	0.37^**^	0.00	0.37^***^

* *p < 0.05*,

** *p < 0.01*,

*** *p < 0.001*.

Note that the Agentic and Communal Values (ACV) scales of agency and communion were positively correlated at *r* = 0.38, *p* < 0.001, which can explain the observed pattern of inter-relationships between the ACCMI and ACV scales, in which Agentic (Communal) Consumer Motivations showed small but significant relationships with Communal (Agentic) Values. By way of comparison, the IPIP-IC scales of Agentic and Communal Traits were uncorrelated with one another, *r* = 0.04, *p* = 0.20, and the patterns of correlations between Agentic (Communal) Consumer Motivations and Communal (Agentic) Traits were much weaker, disappearing entirely for the partial correlations.

We conducted a series of additional analyses in order to compare the magnitude of the relationships between the Agentic and Communal Consumer Motivations scales and the criterion variables. We first compared the relative size of the relationships between the ACCMI scales and the criterion variables. In every case, the Agentic Consumer Motivations scales evidenced larger relationships with the agentic measures than did the Communal Consumer Motivations scales. In every case, the Communal Consumer Motivations scales evidenced larger relationships with the communal measures than did the Agentic Consumer Motivations scales. A sign test revealed that this pattern was statistically significant, *p* < 0.0001. The average difference between the correlations for the agentic criterion variables was 0.34, while the average difference between the correlations for the communal criterion variables was 0.39, corresponding to a medium effect size (Cohen, 1992). Finally, we tested the difference between the magnitude of the correlations of the Agentic and Communal Consumer Motivations scales and each criterion variable using one-tailed tests of the difference between correlations (Steiger, 1980). In every case, including both bivariate and partial correlations, the difference between the Agentic and Communal Consumer Motivations correlations with each criterion variable was statistically significant. In sum, these analyses provide strong evidence for the discriminant validity of the ACCMI scales in predicting the agentic and communal criterion variables.

### Study 1 discussion

In Study 1, we created and refined the bi-lingual ACCMI scale to measure agentic and communal consumer motivations. The resulting 10-item scale evidenced good structural fit across languages, with the MGCFA analyses indicating structural invariance of the measure in its English and French language versions. Furthermore, the ACCMI evidenced good internal consistency and temporal stability across a 3-week time window, and showed good convergent and discriminant validity with a range of motivational and consumer behavior constructs.

The results of Study 1 are consistent with the notion that agency and communion provide a viable framework for studying consumption motivations. This finding serves to link consumption motivations with a larger theoretical and motivational framework, providing a structure through which to understand relevant dimensions of consumer motivation, and underscores the importance of studying agentic and communal motivations conjointly, rather than in isolation from one another. As is the case with a number of other social constructs (traits, values, etc.), agency and communion can provide a rich theoretical perspective from which to understand consumer behavior.

In Study 2, we sought to further demonstrate the validity of the ACCMI measure by showing that it can predict evaluations of consumer products designed to appeal to agentic and consumer motivations.

## Study 2

The goal of Study 2 was to test the predictive validity of the 10-item ACCMI scale in both the English and French language versions. To do so, we created two product descriptions of a generic cell phone which was described in either agentic or communal terms. In a within-subjects design, participants first filled out the ACCMI scales and subsequently evaluated the descriptions of the agentic and communal products.

As a first analysis, we tested the same structural invariance model of the ACCMI presented in Study 1 on the data collected for Study 2. We predicted that, in this new dataset, the ACCMI would evidence satisfactory structural invariance across language versions.

We then conducted a series of analyses to examine the predictive validity of the ACCMI measure. Our primary hypothesis was that Agentic Consumer Motivations would predict evaluation of the agentic product description, while Communal Consumer Motivations would predict evaluation of the communal product description. We further explored whether the predictive validity of the ACCMI scales (e.g., the relationship between motivations and product evaluations) differed between languages. Based on the results of Study 1, we expected that the magnitudes of the relationships between the ACCMI scales and the product evaluations would be equal between the two language versions.

### Method

#### Participants

Anglophone participants were 347 undergraduate students (mean age = 19.77, *SD* = 1.58, due to an experimenter error gender was not assessed) at a large Midwestern American university. Francophone participants were 358 undergraduate students (mean age = 21.06, *SD* = 2.47, 185 males and 173 females) at a large public French-speaking university in the Walloon region of Belgium.

#### Procedure

Participants first completed the 10-item ACCMI. Internal consistencies for the Agentic Consumer Motivations scale were 0.90 and 0.88 in the English and French language versions, respectively. Internal consistencies for the Communal Consumer Motivations scale were 0.90 and 0.81 in the English and French language versions, respectively.

Participants then evaluated two product descriptions for a generic product: a cell phone called the XPhone. Our choice of product was motivated by three factors: (1) cellular telephones are a near universally understood and owned product, particularly among student populations (2) they can convincingly be described as appealing to either agentic or communal motivations, and (3) they have been successfully used in previous research investigating the relationship between individual differences and product evaluation (Hirsh et al., 2012). We created two product descriptions for the generic cellphone (adapted from Hirsh et al., 2012). One product description was designed to appeal to agentic motivations (e.g., XPhone: Stand out from the crowd), and one product description was designed to appeal to communal motivations (e.g., XPhone: Connect with friends and family). This type of product description is often used in market research studies to describe product attributes, positioning, and benefits to consumers (Crawford and Di Benedetto, 2008). The product descriptions were created simultaneously in English and French by a bi-lingual research team, comprising both Native English and Native French speakers. Linguistic equivalency of the product descriptions was verified through back-translation. The English and French language product descriptions are presented in Appendix C. Consistent with previous research on consumer perceptions of commercial communications (e.g., Peck and Wiggins, 2006), participants evaluated the product descriptions on scales designed to assess their attitudes toward the product itself, the description of the product (e.g., the message), and their behavioral intentions toward the product. To measure each of these dimensions, we used the following scales:

##### Product attitude

Participants responded to 3 different 7-point semantic differential items assessing their attitude toward the product (Batra and Stayman, 1990; Peck and Wiggins, 2006). Specifically, participants evaluated the product using the following adjective pairs: Negative-Positive, Unfavorable-Favorable, Bad-Good. Internal consistencies for the agentic product description were 0.92 and 0.95 in the English and French language samples, respectively. Internal consistencies for the communal product description were 0.94 and 0.94 in the English and French language samples, respectively.

##### Attitude toward product description

Participants responded to 3 different 7-point semantic differential items assessing their attitude toward the product description (Batra and Stayman, 1990; Peck and Wiggins, 2006). Specifically, participants evaluated the product description using the following adjective pairs: Negative-Positive, Unfavorable-Favorable, Bad-Good. Internal consistencies for the agentic product description were 0.91 and 0.94 in the English and French language samples, respectively. Internal consistencies for the communal product description were 0.92 and 0.94 in the English and French language samples, respectively.

##### Behavioral intentions

Participants responded to 3 different 7-point semantic differential items assessing their behavioral intentions toward the described product (Taute et al., 2011). Specifically, participants indicated how likely they would be to try each XPhone product using the following adjective pairs: Unlikely-Likely, Definitely Not- Definitely, Improbably-Probably. Internal consistencies for the agentic product behavioral intentions were 0.95 and 0.96 in the English and French language samples, respectively. Internal consistencies for the communal product behavioral intentions were 0.95 and 0.95 in the English and French language samples, respectively.

#### Ethics statement and data availability

The research study was approved in the US by the Institutional Review Board at Michigan State University, No. 12-715e, 2012. In Belgium there is no legal requirement to obtain approval from an institutional review board (IRB) for non-clinical research studies. Two authors of the current paper conducted this research while employed at a Belgian university (Catholic University of Louvain) in which no IRB existed at the time when the data were collected. Belgian participant data were anonymized prior to author access and analysis and no identifying, personal, or health related information was collected. Written informed consent was obtained in the US, and verbal informed consent was obtained in Belgium.

The data from this study are available freely and without restriction at: osf.io/yd3uk.

### Results

#### Cross-language structural validity

Data from Study 2 were subjected to the same two-step MGCFA used in Study 1 to investigate cross-language validity (see Tables 1, 2). Results again suggested structural invariance of the ACCMI across language versions. A test for discriminant validity showed that AVE values for the Agency dimension were 0.63 and 0.61 in the English and French language versions, while the AVE values for the Communion dimension were 0.64 and 0.50 in the English and French language versions. These values are in all cases larger than the squared correlation between the latent factors of these dimensions (0.15 and 0.06 for the English and French language versions, respectively).

#### Predictive validity

Table 4 displays bivariate and partial correlations between the ACCMI scales and the evaluations of the agentic and communal product descriptions. Results for the English and French language samples are presented separately. In both languages, Agentic Consumer Motivations predicted reactions to all three evaluative dimensions for the agentic product description and Communal Consumer Motivations predicted reactions to all evaluative dimensions for the communal product description. Both scales were also discriminant valid for the most part.

**Table 4:** **Bivariate and partial correlations between ACCMI scales and phone evaluation measures by ACCMI language version, study 2**.

**Consumer motivations**	**Correlations**		**Partial correlations**	
	**Agentic**	**Communal**	**Agentic**	**Communal**
**ENGLISH LANGUAGE VERSION**				
**Agentic Phone**				
Product attitude	0.21^**^	0.12^*^	0.21^**^	0.04
Description attitude	0.24^**^	0.08	0.27^**^	−0.02
Behavioral intentions	0.27^**^	0.10	0.28^**^	−0.001
**Communal Phone**				
Product attitude	−0.05	0.20^**^	−0.14^*^	0.27^**^
Description attitude	−0.02	0.17^**^	−0.10	0.21^**^
Behavioral intentions	−0.02	0.17^**^	−0.08	0.20^**^
**FRENCH LANGUAGE VERSION**				
**Agentic Phone**				
Product attitude	0.22^**^	0.11^*^	0.21^**^	0.05
Description attitude	0.20^**^	0.15^**^	0.18^**^	0.11^*^
Behavioral intentions	0.20^**^	0.09	0.19^**^	0.05
**Communal Phone**				
Product attitude	0.08	0.18^**^	0.04	0.18^**^
Description attitude	0.00	0.11^*^	−0.02	0.11^*^
Behavioral intentions	0.01	0.13^*^	−0.02	0.14^*^

* *p < 0.05*,

** *p < 0.01*.

We conducted a series of additional analyses in order to compare the magnitude of the relationships between the Agentic and Communal Consumer Motivations scales and the product evaluation measures. We first compared the relative size of the relationships between the ACCMI scales and the product evaluation measures. In every case, the Agentic Consumer Motivations scales evidenced larger relationships with the agentic product evaluations than did the Communal Consumer Motivations scales. In every case, the Communal Consumer Motivations scales evidenced larger relationships with the communal product evaluations than did the Agentic Consumer Motivations scales. A sign test revealed that this pattern was statistically significant, *p* < 0.0001. The average difference between the correlations for the agentic product evaluations was 0.15, while the average difference between the correlations for the communal product evaluations was 0.20, corresponding to a small-to-medium effect size (Cohen, 1992). Finally, we tested the difference between the magnitude of the correlations of the Agentic Consumer Motivations and Communal Consumer Motivations scales and each criterion variable using one-tailed tests of the difference between correlations (Steiger, 1980). For the raw correlations, 9 of the 12 comparisons were statistically significant. Exceptions were the Agentic Product Attitude in the English language version and Agentic Product Description and Communal Product Attitude in the French language version. For the partial correlations, 11 of the 12 comparisons were statistically significant. The one exception was the Agentic Product Description in the French language version. In sum, these analyses provide strong evidence for the validity of the ACCMI scales in predicting agentic and communal product evaluations.

#### Testing predictive validity across ACCMI language versions

A series of regression analyses was undertaken to determine whether the predictive validity of the ACCMI scales differed between language versions. The primary goal of these analyses was to determine whether the size of the relationships between the ACCMI scales and the product evaluations was equivalent between the English and French ACCMI versions.

In each of these regression analyses, one product evaluation score (e.g., Agentic Product Attitude) served as the dependent variable. Included as independent variables were a dummy variable representing language version (French scored as -1, English as 1), the centered Agentic and Communal Consumer Motivations scale scores, and two interaction terms representing the language version by Agentic Consumer Motivations interaction and the language version by Communal Consumer Motivations interaction. The standardized regression coefficients from these analyses are summarized in Table 5.

**Table 5:** **Multiple regressions of language, motives, and their interaction on agentic and communal product evaluation variables, study 2**.

	**Agentic product evaluation**			**Communal product evaluation**		
	**Product attitude**	**Description attitude**	**Behavioral intentions**	**Product attitude**	**Description attitude**	**Behavioral intentions**
Language	0.21^**^	0.28^**^	0.05	0.17 ^**^	0.22^**^	0.12^**^
Agentic motivations	0.21^**^	0.22^**^	0.23^**^	−0.05	−0.05	−0.05
Communal motivations	0.05	0.05	0.03	0.22^**^	0.15^**^	0.17^**^
Language ^*^ Agentic motivations	−0.01	0.03	0.04	−0.09^*^	−0.04	−0.03
Language ^*^ Communal motivations	−0.02	−0.07	−0.03	0.02	0.02	0.01
Multiple R	0.29	0.34	0.24	0.28	0.27	0.20

* *p < 0.05*,

** *p < 0.01*.

The Agentic Consumer Motivations scale predicted the evaluations of the agentic product in all cases, with standardized betas ranging between 0.21 and 0.23. In no case did the language version by Agentic Consumer Motivations interactions attain significance, indicating that Agentic Consumer Motivations predicted evaluation of the agentic product equally in both languages.

The Communal Consumer Motivations scale predicted the evaluations of the communal product in all cases, with standardized betas ranging between 0.15 and 0.22. In no case did the language version by Communal Consumer Motivations interaction attain significance, indicating that Communal Consumer Motivations predicted evaluation of the communal product equally in both languages.

##### Secondary findings

For most but not all of the dependent variables, we found main effects of language version which revealed that English language participants gave more positive evaluations of both the agentic and communal cell phone product descriptions.

There was one significant language version by motivation interaction in the regression analyses (see Table 5). Specifically, the Agentic Consumer Motivations by language version interaction was significant for the analysis predicting communal product evaluation. Inspection of the correlation matrix in Table 4 indicates that the relationship between Agentic Consumer Motivations and communal product evaluation was weakly negative in the English language version (*r* = −0.05, ns), while this relationship was weakly positive in the French language version (*r* = 0.08, ns). This interaction was not expected and has no clear theoretical interpretation and so it will not be discussed further.

### Study 2 discussion

In Study 2, we further examined the structural and predictive validity of the ACCMI scale. Given that, in Study 1, the same sample was used for the exploratory and confirmatory factor analyses, we re-tested our structural model on the data collected in Study 2. Our analysis revealed that the structure identified in Study 1 provided a good fit to the out-of-sample and out-of-time data from Study 2, with the ACCMI again showing satisfactory structural invariance between the English and French language versions. We then conducted a number of analyses to investigate the predictive validity of the ACCMI across language versions. We first examined the predictive validity of the ACCMI within each language separately, and found in both languages that Agentic Consumer Motivations predicted evaluation of a product described in agentic terms, while Communal Consumer Motivations predicted evaluation of a product described in communal terms. Finally, we tested the equality of the predictive relationships between the ACCMI scales and the product evaluations across language versions. Consistent with our hypotheses, we found that the relationships between Agentic (Communal) Consumer Motivations and evaluations of the agentic (communal) product description were equivalent across languages. In sum, the results of Study 2 provide further evidence for the structural and predictive validity of the ACCMI in its English and French versions.

## General discussion

This research was driven by the assumption that motivational factors are relevant for studying consumer psychology and can be used to better understand buying intentions. Our review of the consumer motivation literature suggested that, as in many other domains (e.g., problems see Horowitz et al., 1988; traits see Markey and Markey, 2009; values see Trapnell and Paulhus, 2012), interpersonal theory can offer a useful framework for understanding motivational factors underlying buying behavior. The results of the current research are consistent with this general hypothesis and show that agency and communion provide useful dimensions which can describe consumer motivations that lead people to prefer certain products over others.

The practical outcome of this research is a bi-lingual measurement instrument, the ACCMI, that can be used to assess motivational determinants of buying behavior. In Study 1, we created a 10-item scale with 5 items measuring Agentic Consumption Motivations and 5 items measuring Communal Consumption Motivations that was reliable, valid, and psychometrically equivalent in English and French. In Study 2, we confirmed the structure of the ACCMI and demonstrated the ability of its agentic and communal scales to uniquely indicate preferences for products described using agentic and communal terms, respectively.

### The application of agency and communion to consumer psychology

At a basic level, this research integrates a general model of human values and social functioning with the specific domain of consumer psychology. Although consumer psychologists and consumer behavior researchers have tended focused on motivational constructs that seem readily describable as agentic (e.g., desire for uniqueness, Tian et al., 2001) or communal (e.g., consumer conformity, Bearden and Rose, 1990), they have mostly done so independently and in a manner that does not recognize the broader agency/communion framework that has been generative in a variety of other domains of psychological research (Wiggins, 1991; McAdams et al., 1996; Smith et al., 1998). While researchers have applied a framework with conceptually similar dimensions to consumer perceptions of brands' intentions (Kervyn et al., 2012), consumer psychologists have not applied a unified framework of this sort toward understanding motivations that drive consumer behavior. In contrast, our approach explicitly places consumer behavior within a basic and general framework of motivation, thereby linking existing consumer research to a rich theoretical system which spans the social sciences. Furthermore, by providing a better understanding of these two consumer motivations and by providing a scale for their measurement from a consumer perspective, the current studies can encourage future research on consumer motivations. The application of interpersonal theory to the specific domain of consumer motivations is consistent with Pham's (Pham, 2013) suggestion that scholars can gain a broadened perspective on consumer behavior by integrating motivational theory and measurement into marketing and consumer psychology research.

### Cross-linguistic scale development

Given our goal of developing an instrument that could be applied broadly, we developed item pools simultaneously in English and French and refined the scale to be valid across language versions. In addition to the enhanced utility of an instrument that can be expected to operate similarly in French and English, it is also possible that items generated and refined in two languages (and cultural contexts) would be more likely to generalize across other languages as well. For instance, such items may be less prone to idiosyncrasies (such as concepts, wordings, etc.) which can influence item performance and selection in mono-linguistic instruments. To the extent that this is the case, this kind of design provides an example for cross-linguistic scale development and may be of particular use in designing measurement instruments for research in multi-lingual countries such as Switzerland, Canada, and Cameroon.

### Applied utility of the ACCMI

The primary applications of this research spring from potential uses of the ACCMI to better understand interpersonal motivations related to consumption behavior. First, consumer motivations can be used to segment consumers into groups, providing insight on consumer market structure according to interpersonal motivations. By understanding agentic and communal consumption motivations among users and non-users of a given category, brand, or product/service, companies can gain insight into motivational factors that can drive consumption behavior. For example, are consumers of luxury cars more agentically motivated in their consumption and how can this insight improve marketing strategy?

Second, understanding interpersonal consumption motivations of one's consumers can help inform product positioning and marketing communication. Interpersonal motivations serve as the basis of many product benefits and as the central message of advertising campaigns, with agentic (e.g., Mountain Dew's “do the Dew” campaign) or communal (e.g., Coca-Cola's “share a Coke” campaign) themes used to position and communicate about products and brands. By better understanding the agentic and communal motivations of consumers of a given category, product or brand, marketers can better position their offer and tailor their advertisements to the motivations of their current or potential consumers.

### Limitations

Several study limitations point to directions for further research. First, study data were collected exclusively in college student samples. While this common sampling frame across populations ensures a degree of similarity among respondents, all of whom are likely to be cell phone users, future research should examine the functioning of the ACCMI scale in different groups. Second, while this study suggests the ACCMI functions satisfactorily in both English and French, future work is needed to test the degree to which its properties generalize across other languages. Third, participants in the current study did not actually purchase products and our study of product evaluations was limited to one product category. Further research should examine actual consumption behavior and purchases, and examine the generalizability of the predictive validity of the ACCMI across product categories. Fourth, we focused in the current research on studying relationships between agency and communion and related constructs via correlation and regression techniques. Future research could fruitfully use Latent Variable Structural Equation Modeling to study the consequences of agentic and communal consumer motivations.

Finally, we focused here on developing efficient indicators of what are likely to be the broadest and most general dimensions of consumer motivations, specifically agency and communion. It is likely that other dimensions are relevant for understanding buying motives. For instance, analyses of our original 90 items provided some evidence for a group of items implying conformity. This is to be expected, given that our literature review and item development process was informed by research on consumer conformity. Ultimately, however, this component was not pursued as it was not consistent across languages or well-represented in our item pool. It is worth noting that, even though conformity motivations are not explicitly tapped by the final ACCMI items, the results of Study 1 show that the Communal Consumer Motivations scale of the ACCMI shows convergent validity with conformity motivations (as indexed by the ATSCI scale, raw and partial correlations of 0.31 and 0.36, respectively). There are likely to be other motivational dimensions that are relevant to understanding consumer behavior and which are not explicitly tapped by the ACCMI. Future research should more thoroughly explore the relationship between various aspects of consumer motivations; the current study and the ACCMI in particular provide a solid initial foundation for such work.

## Conclusions

This research suggests the importance of understanding fundamental motivations related to consumer behavior. We use agency and communion as a guiding framework for the study of consumption motivations, and develop a bi-lingual measure of agentic and communal consumer motivations, the ACCMI, which can be used in studies among English and French-speaking populations. Given the relevance of motivations for understanding consumption behavior, it is our hope that the ACCMI measure will help stimulate the further integration of motivational theory (including agentic and communal motivations) into the marketing and consumer psychology literature.

## Author contributions

MF conceived and designed the research studies, collected data, conducted data analysis, and co-wrote the paper. AB conceived and designed the research studies, collected data, conducted data analysis, and co-wrote the paper. JL conceived and designed the research studies and collected data. CH conceived and designed the research studies, collected data, conducted data analysis, and co-wrote the paper.

### Conflict of interest statement

The authors declare that the research was conducted in the absence of any commercial or financial relationships that could be construed as a potential conflict of interest.
