# Supplementary material for: The Development of a Bi-Lingual Assessment Instrument to Measure Agentic and Communal Consumer Motives in English and French
Source: Front Psychol. 2016 Aug 11;7:1198. doi: 10.3389/fpsyg.2016.01198 (PMC4980988; doi:10.3389/fpsyg.2016.01198)
Supplement: Supplementary file 1 [file DataSheet1.docx]

**Appendix A: Agentic and Communal Consumption Motivation Scales: English Version**

People buy products for many reasons (e.g. price, quality, etc.). We are interested in purchases that help people to express their inner values, to show the world “who they are.” The questions below ask about this issue. Please indicate your response to each question using the following scale:

| 1 | 2 | 3 | 4 | 5 | 6 |
| --- | --- | --- | --- | --- | --- |
| Not important to me | Mildly important to me | Somewhat important to me | Moderately important to me | Very important to me | Extremely important to me |

For each item below, please answer the following question:

***How important is it to me that the products I buy, in general, help me to:***

1. be unlike others

2. be uncommon

3. seek harmony with others

4. stand out from others

5. be different

6. seek connection with others

7. belong

8. be original

9. seek unity with others

10. pay attention to others

**Scoring Key:**

**Agentic Consumer Motivations: Item 1, Item 2, Item 4, Item 5, Item 8**

**Communal Consumer Motivations: Item 3, Item 6, Item 7, Item 9, Item 10**

**Appendix B: Agentic and Communal Consumption Motivation Scales: French Version**

Les personnes achètent des produits pour plusieurs raisons (ex: prix, qualité,...). Nous sommes intéressés par les achats qui aident les personnes à exprimer leurs valeurs intérieures, afin de montrer au monde "qui elles sont". Les questions ci-dessous traitent de ce sujet.

Merci d'indiquer votre réponse à chaque question en utilisant l'échelle d'évaluation qui suit :

| 1 | 2 | 3 | 4 | 5 | 6 |
| --- | --- | --- | --- | --- | --- |
| Pas important pour moi | Peu important pour moi | Assez important pour moi | Modérément important pour moi | Très important pour moi | Extrêmement important pour moi |

Pour chaque item ci-dessous, merci de répondre à la question suivante :

**Dans quelle mesure est-il important pour moi que les produits que j’achète, en général, m’aident à :**

1. ne pas ressembler aux autres

2. être hors du commun

3. rechercher l'harmonie avec les autres

4. me démarquer des autres

5. être différent(e) des autres

6. rechercher une connexion avec les autres

7. m'intégrer

8. être original(e)

9. rechercher l’union avec les autres

10. être attentif(ve) aux autres

**Scoring Key:**

**Agentic Consumer Motivations: Item 1, Item 2, Item 4, Item 5, Item 8**

**Communal Consumer Motivations: Item 3, Item 6, Item 7, Item 9, Item 10**

**Appendix C: Agentic and Communal Product Descriptions, Study 2**

**English Versions**

*Agentic Product Description*

**XPhone: Stand out from the crowd**. This cellphone is designed to make you stand out from the crowd. With the new XPhone, dare to be different and proclaim your independence. Designed to be both stylish and unique, the XPhone is ideal to differentiate yourself from others. With the XPhone, embrace your original and unconventional side.

*Communal Product Description*

**XPhone: Connect with friends and family**. This cellphone is designed to bring people together. With the new XPhone, you can always be in touch with your community. Designed with empathy and consideration, the XPhone helps you connect with the people you care most about. With the XPhone, find out where you belong.

**French Versions**

*Agentic Product Description*

**XPhone: Démarquez-vous.** Ce GSM est conçu pour vous démarquer. Avec le nouveau XPhone, osez être différent(e) et clamez votre indépendance. Conçu pour être à la fois stylé et unique, le XPhone est idéal pour vous différencier des autres. Avec le XPhone, assumez votre côté original et non conventionnel.

*Communal Product Description*

**XPhone: Rester connecté(e) avec vos amis et votre famille.** Ce GSM est conçu pour rassembler les gens. Avec le nouveau XPhone, vous pourrez toujours être en contact avec votre entourage. Conçu avec de l’empathie et de la considération, le XPhone vous aide à rester connecté(e) avec les personnes qui comptent le plus pour vous. Avec le XPhone, trouvez votre place.
